# Systematic Review of Commercially Available Mobile Phone Applications for Prostate Cancer Education

**DOI:** 10.1177/1557988318816912

**Published:** 2018-12-07

**Authors:** Otis L. Owens, Jenay M. Beer, Ligia I. Reyes, Tracey L. Thomas

**Affiliations:** 1 College of Social Work, University of South Carolina, Columbia, SC, USA; 2 College of Public Health, University of Georgia, Athens, GA, USA; 3 Department of Health Promotion Education and Behavior, Arnold School of Public Health, University of South Carolina, Columbia, SC, USA; 4 Health Sciences Department, Health and Behavioral Studies College, James Madison University, Harrisonburg, VA, USA

**Keywords:** prostatic neoplasms, decision making, early detection of cancer, mobile applications, review

## Abstract

Prostate cancer is the most commonly diagnosed non-skin cancer among all men and the second most common cause of death. To ameliorate the burden of prostate cancer, there is a critical need to identify strategies for providing men with information about prostate cancer screening and the importance of informed decision making. With mobile phones becoming more ubiquitous, many individuals are adopting their phones as sources for health information. The objective of this systematic review is to identify and evaluate commercially available apps for promoting informed prostate cancer screening decisions. Two keywords “prostate cancer screening” and “prostate cancer” were entered into the search engines of Google and iOS app stores in May 2017. Evaluations were conducted on apps’ (a) quality, (b) grade-level readability, (c) cultural sensitivity, and (d) usability heuristics. None of the 14 apps meeting the inclusion criteria contained the full breadth of information covered in the 2016 American Cancer Society’s Prostate Cancer Prevention and Early Detection Guidelines, but over half were inclusive of topics consistent with these guidelines. Most apps’ readability was higher than an eighth-grade reading level. Most apps were also not framed and had a neutral tone. Only four apps met most criteria for being culturally sensitive to African Americans. Usability among apps was variable, but some contained major usability concerns. Recommendations for improving educational apps for prostate cancer screening include: disseminating evidence-based information; using culturally sensitive language; knowing the implications of the one and framing of content; making apps interactive; and following common usability principles.

Prostate cancer (PrCA) is the most commonly diagnosed non-skin cancer among all men and the second most common cause of death (Siegel, Miller, & Jemal, 2018). PrCA is the most detrimental among African American men, who are twice as likely as their White counterparts to experience mortality from the disease (Siegel et al., 2018). To reduce the burden of PrCA among all men, there has been fervent controversy about the routine use of the prostate specific antigen (PSA) screening as a prevention measure (Carlsson et al., 2012; Catalona, 2012). Most of the debate regarding the PSA exam is based on evidence that it can lead to false negatives and the identification of indolent PrCA (which constitute over 80% of all PrCAs; Barry, 2009; Friedrich, 2011). There is also a lack of evidence regarding the predictive validity of the digital rectal exam, which has led some researchers to also recommend against routine PrCA screening using this method (Naji et al., 2018) Despite their diverging ideas about the efficacy of PSA screening and the digital rectal exam (i.e., PrCA screening), most organizations, including the American Urological Association (AUA), American Cancer Society (ACS), and the United States Preventive Services Task Force (American Cancer Society, 2016; Bibbins-Domingo, Grossman, & Curry, 2017; Carter et al., 2013), recommend that men make an informed decision with their health-care provider about whether to receive PrCA screening. According to the Centers for Disease Control and Prevention, informed decision making (IDM) is when a man understands a condition; the risk, benefits, alternatives and uncertainties of clinical intervention; and participates in a decision at the level desired (Rimer, Briss, Zeller, Chan, & Woolf, 2004). The AUA and the United States Preventive Services Task Force recommend that men at an average risk for PrCA should make informed PrCA screening decision between 55 and 69, while the ACS recommends conversations for these men begin at age 50. For men at high risk for PrCA (such as those who are African American or have a family history of the disease), the AUA recommends that conversations about PrCA screening begin as early as age 40 while the ACS recommends age 45 (American Cancer Society, 2016; Bibbins-Domingo et al., 2017; Carter et al., 2013).

To make an informed PrCA screening decision, Healthy People 2020 and the Institute of Medicine support the use of effective, readable, and culturally sensitive cancer communication strategies (Institute of Medicine, 2004; U.S. Department of Health and Human Services, 2011). With mobile phones becoming more ubiquitous, many individuals are adopting their phones as sources for a health information. Over 77% of adults own a smartphone (Pew Internet & American Life Project, 2017) and over half of these users download health-related apps (Krebs & Duncan, 2015). Because there are few regulations on health-related apps, the quality of the information in these apps is debatable (Barton, 2012). Quality in this study refers to an apps’ accuracy (whether the health content provided in the app is correct), breadth (the extent to which the app content includes all pertinent details), grade-level readability (the level of education one must have attained to read an English passage with ease; Friedman & Hoffman-Goetz, 2006), cultural sensitivity (the extent to which interventions incorporate cultural characteristics, norms, values, and beliefs; Resnicow, Baranowski, Ahluwalia, & Braithwaite, 1999), and usability (the degree to which a user interface, such as an app, is easy to use; Nielsen, 1994). Each of these measures can affect whether a man gains adequate knowledge to effectively engage in IDM.

In addition to app quality, the framing of PrCA information is also critical for influencing IDM. Framing is the mechanism by which the media select certain aspects of perceived reality and make them more salient to the receiving audience (Entman, 1993). Following their receipt of these frames, the audience comprehends, judges, and makes inferences about the world (Scheufele & Iyengar, 2012). Therefore, the way health problems and solutions are framed can impact the public’s understanding of a health topic.

One method for framing health risk information, such as cancer, is through gain and loss framing. Gain-framed messages “emphasize the desirable consequences of compliance with the advocated view” while loss frame messages “emphasize the undesirable consequences of noncompliance” (O’Keefe & Jensen, 2007). Gain/loss framing closely relates to prospect theory, which suggests that people’s decisions are influenced by whether a message emphasizes benefits or costs (Ganegoda & Folger, 2015).

Multiple scientists have examined the effects of the gain and loss framing as they specifically relate to the benefits and risks of cancer screening (Consedine, Horton, Magai, & Kukafka, 2007; O’Keefe & Jensen, 2007, 2008; Thomas et al., 2011). Gallahgher, Updegraff, Rothman, and Sims (2011) reported that women with perceived susceptibility for breast cancer were more likely to report having received a mammogram following the receipt of a loss-framed message as opposed to a gain-framed message. While there is some mixed evidence regarding whether loss-framed messages or gain-framed messages are more pervasive on the prevention behaviors of individuals (Cho & Sands, 2011; Edwards, Elwyn, Covey, Matthews, & Pill, 2001; Jones, Sinclair, & Courneya, 2003), most researchers have reported that loss-framed messages may be more likely to influence health behaviors (Gallagher & Updegraff, 2011; Gallagher, Updegraff, Rothman, & Sims, 2011). If an app depicts the negative consequences of not obtaining PrCA screening, it may cause an individual to proactively seek screening. Whereas, when presented with benefits of PrCA screening, an individual may be less likely to seek screening. Despite the framing, apps could prove harmful if men are not informed about the risks of screening and do not engage in IDM.

Overall, the growing interest in the receipt of health and cancer information through apps and the lack of regulation over these apps raises concerns regarding the quality and framing of PrCA information and to what extent apps prepare men to engage in IDM with providers. In particular, the purpose of our study is to conduct a systematic review of Android and iOS apps to determine: (a) the quality of PrCA content based on the accuracy, breadth, grade-level readability, and cultural sensitivity of the app content; (b) whether the information regarding PrCA screening is framed as a gain, loss, or neutral; and (c) to what extent the app meets validated usability standards. Though African Americans are not the sole focus of this review, we have given attention to the cultural sensitivity of PrCA apps because of the high mortality among this population (Siegel et al., 2018).

## Methods

### Keywords and App Search

To conduct this review, the keywords “prostate cancer screening” and “prostate cancer” were entered into the search engines for the Google (i.e., Android) and iOS app stores between April and May 2017. A database was developed to record search results, documenting the keywords used, date of searches, number of app results, and app names. All apps were selected and reviewed via the process described below.

### Inclusion and Exclusion Criteria

**Figure 1.**
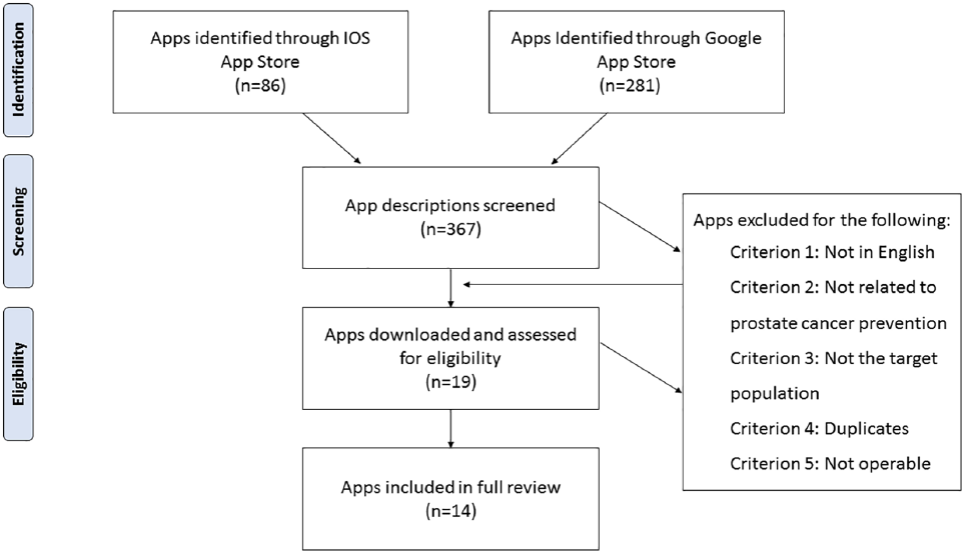
App inclusion flow diagram.

For the inclusion criteria, the approach used by Coulon, Monroe, and West (2016) was adapted. Specifically, app descriptions were reviewed to determine whether it (a) was available in English, (b) included general information about PrCA prevention, and (c) targeted the general population, not health professionals. Apps were excluded if they were not in English; targeted health professionals; functioned as a symptom tracker or symptom calculator; served as a channel/gateway to other services; or exclusively targeted treatment, survivorship, or social support mechanisms for adults already diagnosed with PrCA. Apps that were duplicates either within a single app store or across stores were also excluded. If apps were duplicated across stores, the iOS app was retained for review because of the lower proportion of apps in the iOS store compared to the Google app store. When inclusion criteria could not be determined from the app description, the app was downloaded for further assessment. The search resulted in 281 apps in the Google app store and 86 apps in the iOS store. Of these apps, 11 Google and 8 iOS apps met the inclusion criteria for full evaluation. Four of the 11 Google and one iOS apps were inoperable or removed by the time of the full evaluation, leaving 14 apps for the final review. Both paid and free apps were included in this review. See Figure 1 for more details. All apps included in our review were released onto the app market after 2015, which proceeded all of the latest PrCA screening recommendations at the time of the review.

### Evaluation Measures and Process

#### Accuracy and breadth

A content evaluation codebook was developed using the 2016 ACS Prostate Cancer Prevention and Early Detection Guidelines (American Cancer Society, 2016). These guidelines were selected for the codebook because they were developed for lay individuals. ACS is one of the most respected authorities on cancer prevention and control. The content was assessed on 10 primary questions (see Table 1) about prostate location and function, PrCA prevalence and risk factors, and PrCA screening tests. Each primary question enabled the rater to indicate whether an app included information about a given area. For example, one question asked, “Does the app provide information about the location of the prostate?” When the primary question had an affirmative response, it prompted two follow-up questions about the accuracy and breadth of the information. For ease of rating accuracy and breadth, key points from the ACS Prostate Cancer Prevention and Early Detection Guidelines were listed on the evaluation form. When there was a negative response regarding the accuracy or breadth of the information, a rater was prompted to elaborate. All scores were recorded in Google forms, an online resource that enables users to populate scores from multiple raters.

**Table 1.** Prostate Cancer Content Questions.

Question number	Question text	Example key points
1	Does the app provide information about the location of the prostate?	The prostate is a gland part of the male reproductive system.
2	Does the app provide information about the function of the prostate?	The prostate produces some of the fluid in semen.
3	Does the app provide information about the prevalence and incidence of PrCA?	About 1 in 7 men is diagnosed with PrCA during his lifetime.
4	Does the app provide information about the risks for PrCA?	African American men are more than twice as likely than White men to die from PrCA. PrCA is less prevalent among Asian-American men and Hispanic/Latino men when compared to their White counterparts.
5	Does the app provide information about the recommended screening age?	Screening age is 45 for men at high risk, including African American men and men who have a first-degree relative, especially if diagnosed at a younger age.
6	Does the app provide information about the symptoms of PrCA?	It is uncommon for early stages of PrCA to cause symptoms and advanced stages only sometimes cause symptoms, which include: urinary problems, erectile dysfunction, etc.
7	Does the app provide information about the digital rectal exam?	The digital rectal exam does not have 100% accuracy and can have both false-positive and false-negative results.
8	Does the app provide information about the PSA test?	PSA stands for Prostate-Specific Antigen blood test, which is sensitive to other factors and therefore not 100% accurate.
9	Does the app provide information about PSA levels related to the probability of having PrCA?	Men with PSA levels 4 < 10 have about 25% chance of having PrCA.
10	Does the application discuss the controversy behind PSA screening?	Not everyone agrees that PSA screening should be performed on an annual basis.

To establish intercoder reliability (Hunt, 1986), two raters evaluated seven iOS apps (50%) using a mobile device. iOS apps were selected for this calculation because both raters owned iOS devices. Percent agreement was calculated by dividing the total number of agreements by the total possible items that could be rated. The initial percent agreement between raters was 76.67%. After meeting to discuss inconsistencies in ratings, seven apps were re-rated and percent agreement was recalculated at 98.84%. The second rater then independently evaluated the remaining seven Android apps.

#### Tone and framing

The tone of each app was measured as a global assessment of the app’s position on the PrCA screening controversy. More specifically, apps evaluated based on whether the content was pro-screening (i.e., encourages men to screen), anti-screening (i.e., discourages men from screening), or neutral (i.e., neither discourages nor encourages screening). Apps were also examined for gain and loss frames. Gain-framed messages emphasized the benefits of screening and loss frames emphasized cost of not being screened. Each app was rated using four categories: gain-framed apps; loss-framed apps; mixed-framed apps (i.e., mixture of gain- and lose-framed messages); and non-framed apps. To establish intercoder reliability, percent-agreement was calculated based on two raters’ evaluation of the seven iOS apps. The percent agreement between raters was 100%.

#### Grade-level readability

Readability was evaluated using Readibility.io (Readability.io, 2018), a usability software that has been used in similar research (Solovyev, Ivanov, & Solnyshkina, 2017). Readability.io provides grade-level scores according to five standardized reading scales including Flesch-Kincaid Grade Level, Gunning Fog Score, Coleman-Liau Index, SMOG Index, and Automated Readability Index (Friedman & Hoffman-Goetz, 2006) along with an average of the five scores. Readability scores of each app were determined using 150 words of text.

#### Cultural sensitivity

To determine the cultural sensitivity of the apps for African Americans, the Cultural Sensitivity Checklist (Friedman & Hoffman-Goetz, 2006) was adapted. Cultural sensitivity among African Americans was included in the study because this population has incidence and mortality rates that are two to three times higher than their White counterparts (Siegel et al., 2018). Further, there is a general paucity of culturally sensitive cancer PrCA information for African Americans available through health-care provider offices (Choi et al., 2018) or through interventions such as mobile or computer-based decision aids (Stacey et al., 2017), though it has been reported that having access to culturally sensitive information may lead to better outcomes among African Americans (Tucker et al., 2014). The Cultural Sensitivity Checklist was designed to evaluate printed material, but has also been used for online material (Friedman & Kao, 2008). The original checklist contained eight items, of which six were pertinent to the study. The remaining two items overlapped with the readability evaluation. Each app was scored on whether it met, somewhat met, or did not meet the Cultural Sensitivity Checklist criteria. To establish intercoder reliability, two raters conducted separate evaluations for half of the apps (seven iOS apps) and reached 100% agreement. One reviewer rated the remaining seven Android apps.

#### Usability

An expert in human–computer interaction developed the usability heuristics evaluation questionnaire based on Nielsen’s 10 usability heuristics (Nielsen, 1994; Pierotti, 1995; Yáñez Gómez, Cascado Caballero, & Sevillano, 2014; see Table 2). Two raters independently evaluated usability heuristics for all apps using Google forms. Each rater then crafted a justification statement for apps that violated any respective heuristic. The two raters then compared their ratings/comments and came to a consensus on which violations were most prominent across apps.

**Table 2.** Usability Heuristics for User Interface Design.

Heuristic^a^	Definition	Questionnaire items
Visibility	The system should always keep users informed about what is going on, through appropriate feedback within a reasonable amount of time.	“Does every screen begin with a title or header?” “It is obvious to the user what is going on?” “Is the font large enough?”
Match between system and real world	The system should speak the users’ language, with words, phrases, and concepts familiar to the user, rather than system-oriented terms. Follow real-world conventions, making information appear in a natural and logical order.	“Are menu choices and information ordered in a logical way?” “Do related and interdependent information appear together?” “Is language clear and concise (terminology familiar to users)?”
Consistency	Users should not have to wonder whether different words, situations, or actions mean the same thing. Follow platform conventions.	“Does the app use a minimal number of colors (i.e., color consistency)?” “Is there a consistent design scheme across the app?” “Do online instructions/information appear in a consistent location across screens?”
User control and freedom	Users often choose system functions by mistake and will need a clearly marked “emergency exit” to leave an unwanted screen without having to go through an extended dialogue. Support “undo” and “redo” actions.	“Is there navigation on the homepage of the app?” “Can users easily reverse their actions?” “Is the app explore-able and easy to navigate?”
Error prevention	Even better than good error messages is a careful design that prevents a problem from occurring in the first place. Either eliminate error-prone conditions or check for them and present users with a confirmation option before they commit to an action.	“Are menu choices logical, distinctive, and mutually exclusive?” “Are buttons/commands placed a good distance from one another?” “Does the system prevent users from making errors whenever possible?”
Recognition rather than recall	Minimize the user’s memory load by making objects, actions, and options visible. The user should not have to remember information from one part of the dialogue to another. Instructions for use of the system should be visible or easily retrievable whenever appropriate.	“Are instructions visible?” “Is it obvious what is clickable?” “Does the app require high levels of concentration?”
Flexibility and efficiency of use	Accelerators—unseen by the novice user—may often speed up the interaction for the expert user such that the system can cater to both inexperienced and experienced users. Allow users to tailor frequent actions.	“Does the app provide function keys for high-frequency commands?” “Does the app allow for customization (e.g., settings, search)?” “Does the app provide customization for frequency users (e.g., log in, saves data)?”
Aesthetic and minimalist design	Dialogues should not contain information that is irrelevant or rarely needed. Every extra unit of information in a dialogue competes with and diminishes the visibility of relevant information.	“Is the layout clearly designed avoiding visual noise?” “Does the use of images and multimedia content add value?” “Are images well sized and is the resolution appropriate?”
Error recovery	Error messages should be expressed in plain language (no codes), precisely indicate the problem, and constructively suggest a solution.	“Are there error messages?” “Is sound, images, or haptics used to signal an error?” “Are error messages worded so the user understands the problem and what to do next?”
Help and documentation	Ideally, the system can be used without documentation, but in the case of questions or confusion, it’s important to provide help and documentation. Any such information should be easy to search, focused on the user’s needs, list concrete steps to be carried out, and not be too lengthy.	“Are there instructions/help/documentation?” “Are navigation and instructions easy to find?” “Are navigation and instructions procedural (how do I use the app)?”

## Results

### Accuracy and Breadth

Of the 14 apps reviewed, 12 apps (86%) contained information about the prostate’s location. These apps contained accurate information and 11 of these 12 apps (92%) included the full breadth of information covered in the 2016 ACS Prostate Cancer Prevention and Early Detection Guidelines. Twelve apps (86%) also provided full and accurate information about function of the prostate. In addition, 12 apps (86%) provided accurate information about the prevalence and incidence of PrCA, but three of these apps (25%) did not include the full breadth of information about this topic. Eleven apps (79%) included accurate information about the risks for PrCA, but only two of these apps (18%) included the full breadth of information. Furthermore, 11 apps (79%) included accurate information about select symptoms for PrCA, while seven of these apps covered the full gamut of symptoms.

Nine of the 14 apps (64%) reviewed included information about the age men should be screened for PrCA, and two of these apps (22%) did not cover the topic thoroughly. Eight apps (57%) included accurate information about the digital rectal exam (a screening for PrCA) and all but one of these apps (87%) included a comprehensive presentation of the topic. A total of 12 apps (86%) included accurate information about the PSA exam, but only seven of these apps (14%) fully covered the topic. In addition, six apps (43%) provided accurate information about what numbers constitute healthy PSA levels, four (67%) of which included comprehensive information on this topic. Lastly, only five of the 14 apps (36%) reviewed mentioned the controversy regarding the efficacy of the PSA exam. All but one of these five apps (80%) provided adequate detail. See Table 3 for additional information.

**Table 3.** Results.

App name	Developer	PrLo^c^	PrFu^d^	Prev^e^	Risk^f^	Age^g^	Symp^h^	DRE^i^	PSA^j^	PSA2^k^	Cont^l^
iURO Oncology^a^	CommunityToGo Pty Ltd	I						I	I		
PCFA Know Your Score WA^a^	CommunityToGo Pty Ltd			I		I	I		I		
Best Prostate Cancer Treatment^a^	RL Technology, LLC	F	F	F	F	F	F	F	F	F	F
Cancer Research News & Prevention Info^a^	Juicestand Inc				I				I		F
My Prostate Health Navigator^a^	Sourcetoad, LLC	F	F	F	I		I	F	F	I	I
Prostate Cancer Treatment and Prevention^a^	Monica G	F	F	F	I		I		I		
Prostate Cancer^a^	Focus Medica India Pvt. Ltd	F	F	F		I		F	I	I	
300 Tips to Prevent Cancer (i.e., Oncotip)^b^	Let ME Hear Again Apps	F	F	F	I	F	F	F	F	F	
Zero Prostate Cancer News^b^	Fuzz Labs	F	F	F	F	F	F	F	F	F	F
itsaMANTHING	PROSTaid	F	F	F	I	F	F		F		
Prostate Cancer^b^	Anastore	F	F	F	I	F	F		I		
Prostate Cancer^b^	Magna Health Solutions	F	F	F	I	F	F	F	F	F	
Cancer Awareness^b^	Surendrasinh Champavat	F	F	F	I	F	F				
PROCEE^b^	Interactive Systems Research Group	F	F	I	I	F	I	F	F	I	F

*Note.* An app containing full information on the specified topic is designated by a C, an app containing incomplete information is indicated by an F, and an app containing no information is indicated by a blank cell.

a iOS app, ^b^Android app, ^c–l^questions used for our content review including whether an app included information about the: ^c^location of the prostate, ^d^function of the prostate, ^e^prevalence and incidence of PrCA, ^f^risks for PrCA, ^g^recommended screening age, ^h^symptoms of PrCA, ^i^digital rectal exam (DRE), ^j^PSA test, ^k^PSA levels related to the probability of having PrCA, and ^l^controversy behind PSA screening.

### Tone and Framing

All but one app (93%) had a neutral tone when presenting PrCA screening information, with only one app (7%) using pro-screening language and no apps using anti-screening language. The neutral tone apps typically included a brief fact-based summary of the PSA and digital rectal exams but either did not discuss pros and cons of screening or did so in a balanced way. In contrast, the pro-screening app discussed only the benefits of screening, focusing primarily on the importance of early detection.

Of the 14 apps reviewed, nine apps (65%) were non-framed. Three apps (21%) were gained framed, with these apps focusing on the benefits associated with knowing one’s PrCA status. The final two apps (14%) were mixed framed and aggregated current PrCA news from multiple sources, with some of the sources including gain frames and others using loss frames in screening discussions. None of the apps employed a loss frame.

### Grade-Level Readability

On average, apps contained content at the tenth grade level. Four apps (29%) had an eighth-grade readability level or below. Five apps (36%) had grade-level readability levels that were between ninth grade and college-level. Readability scores could not be calculated for four apps (29%) because they did not contain enough text to calculate a readability score. All of these apps provided PrCA education through videos. One remaining app (7%), Prostate Cancer Treatment and Prevention, was no longer available on the app store when readability scores were calculated (see Table 4).

**Table 4.** Grade-Level Reading Scores.

App name	Grade-level readability score
iURO Oncology	Unable to calculate
PCFA Know Your Score WA	9.6
Prostate Cancer & Colon Cancer	8.3
Cancer Research News & Prevention Info	Unable to calculate
My Prostate Health Navigator	8.3
Prostate Cancer Treatment and Prevention	Unable to calculate
Oncotip	12.8
Zero Prostate Cancer News	16
itsaMANTHING—Prostate Cancer	9.5
Prostate Cancer (Anastore)	8.2
Prostate Cancer (Magna Health)	7.8
Cancer Awareness	9.0
Prostate Cancer (Focus)	Unable to calculate
PROCEE	Unable to calculate

### Cultural Sensitivity

Five of the 14 apps (36%) did not meet any Cultural Sensitivity Checklist criteria. Five additional apps (36%) met or somewhat met one to three of these criteria. Three apps (21%) met four to five of Cultural Sensitivity Checklist criteria. The remaining app, Prostate Cancer Treatment and Prevention, could not be rated because it was no longer available at the time of rating. The criteria least often met were (a) the contact person or organization that developed the app was African American or that caters to African Americans (*n* = 1), followed by (b) the information addresses the perceptions of cancer risk in among African Americans (*n* = 2). The criteria that were met most often (*n* = 7) related to whether African Americans were described as a high-risk group for cancer. Additionally, seven apps were also linked to credible sources.

## Discussion

### Overview

This app review is the first to explore the quality, framing, and usability of commercially available PrCA prevention apps on the Android and iOS markets. Of the 14 apps evaluated, 11 apps (79%) focused on providing general PrCA education through text only or video only formats. One app called Procee, provided an interactive educational experience where animated characters engaged in a PrCA-related dialogue and asked users to answer questions to tailor the user’s educational experience. The two remaining apps, Cancer Research News & Prevention Info Free and Prostate Cancer - News/Videos, primarily provided news content relevant to PrCA prevention. All but one of the apps (Procee, developed for African American men) did not appear tailored for a specific race. Also, most apps were free with exception to Prostate Cancer by Focus Medica (cost: $7.99), Prostate Cancer Treatment and Prevention (cost: $2.99), and Prostate Cancer Guidelines (cost: $0.99). Furthermore, app costs did not correlate with app performance. In particular, neither of the apps that bared a cost ranked higher than free apps in accuracy or usability.

Only one app evaluated included the full breadth of information covered in the 2016 ACS Prostate Cancer prevention and Early Detection Guidelines (Best Prostate Cancer Treatment), but only because the app linked directly to the ACS Early Detection Guidelines webpage. However, over half of the apps were at least inclusive of topics consistent with ACS guidelines. In addition, some topics were more likely to include the appropriate breadth of information than others. Specifically, most apps included information about prostate anatomy and almost all apps included the full breadth of details on this topic. Similarly, 12 apps included information about the prevalence of PrCA, and 10 of these apps were thorough in their description of the topic.

A second subset of topics including (a) age at which men should discuss screening with their health-care provider, (b) the digital rectal exam, and (c) the controversy of the PSA exam was mentioned in five to eight apps and the majority of the apps covered these topics thoroughly. For example, eight of nine apps that included information about recommended ages that men should discuss PrCA screening also indicated that this age is dependent on factors relevant to an individual’s risk for PrCA such as their race, as opposed to providing a catch-all age at which all men should be screened. The remaining topics, including PrCA risks, PrCA symptoms, and the PSA exam were mentioned in 11 to 13 apps but rarely provided adequate detail. For example, while age and race were commonly indicated as risk factors for PrCA, only two apps, Zero Prostate Cancer News and Best Prostate Cancer Treatment, provided details about the contribution of family history, genetics, and other hypothesized risk factors such as diet, obesity, smoking, chemical exposure, prostate inflammation, STIs, and so forth.

Knowing information about PrCA risks and symptoms (particularly the fact that PrCA rarely has symptoms) and the age at which discussions about screening should begin may be highly influential to whether a man engages in an informed decision with their health-care provider about PrCA screening. Of equal importance to the IDM process is men’s knowledge about the types of screenings available and thorough details about the risks, benefits, and uncertainties of these screenings, which includes the controversy regarding the efficacy of the PSA screening (Kim & Andriole, 2015). Though less invasive than the digital rectal exam, the PSA has long been debated as a test that should not be used as a routine screening to detect PrCA (Ablin, 2010; Barry & Patient Outcomes Research Team for Prostatic Diseases, 1998; Brett & Ablin, 2011). These reservations are a result of the test’s sensitivity and the likelihood that it can lead to the detection and treatment of indolent cancers (Hoffman, 2011; Manley & Andriole, 2016). Being informed about this controversy can be especially advantageous in scenarios where a health-care provider fails to mention the risks of the PSA exam. In particular, Bhuyan et al. (2017) identified through a national survey of 1,706 men that health-care providers communicate with patients about the PSA controversy 17.2% of the time and about the test’s accuracy 25.4% of the time.

Overall, the apps that provided the most thoroughly covered PrCA content relative to the 2016 ACS Prostate Cancer Prevention and Early Detection Guidelines were Best Prostate Cancer Treatment, Oncotip, and Prostate Cancer by Magna Health Solutions. Best Prostate Cancer Treatment was linked directly to ACS’s website. Oncotip Cancer Prevention included a plethora of PrCA information, but lacked only a few risk factors (e.g., geography) and did not provide information on the PSA controversy. Prostate Cancer by Magna Health Solutions also included ample content but did not mention race as a key risk factor and lacked information about the PSA controversy.

In general, the tone used in apps to present screening information was neutral, with all but one app discussing screening in a factual and balanced way. The only app that was pro-screening, particularly focusing on the importance of early detection and diagnosis with respect to improved treatment options, was Oncotip. This was also only one of three apps that used a gain-frame when discussing PrCA screening. The other two gain-framed apps (Oncotip and Prostate Cancer by Anastore) were neutral in tone even though they focused on the benefits associated with screening. Still, the majority of apps were non-framed, primarily resulting from the brevity of discussions about PrCA screening, with apps typically providing only brief fact-based summaries of common screening exams and offering no or very limited commentary. The lack of gain/loss framing may suggest that the majority of the apps will have limited influence on users’ decisions related to PrCA screening. The absence of loss-framed screening messages in particular may limit the influence apps have on users’ screening decisions, as previous research suggests that loss-framed messages may be more likely to influence behaviors such as cancer screening when compared to gain-framed messages (Gallagher & Updegraff, 2011; Gallagher et al., 2011).

The average grade-level readability for apps in the review was 10th grade, which is about two grades above the standard used by the American Medical Association (American Cancer Society, 2016) and agencies within the U.S. Department of Health and Human Services (Stableford & Mettger, 2007). These findings are consistent with similar app review that assessed the readability of apps for lung disease (Owens et al., 2018). Prostate Cancer by Magna Health Solutions, which scored a 7.8, had the lowest readability, and Zero Prostate Cancer News, which scored a 16 (college level), had the highest. The Zero Prostate Cancer News app not only features information about general PrCA information but also PrCA research which may have inflated the grade-level readability. Though grade-level readability is not synonymous with health literacy (i.e., the degree to which individuals have the capacity to obtain, process, and understand basic health information; *National Action Plan to Improve Health Literacy*, 2010), writing health information at the appropriate grade level can make PrCA information more accessible to individuals with lower education levels.

Four apps met most of the criteria for being culturally sensitive to African Americans. In addition, study findings regarding the low overall cultural sensitivity for African Americans among most apps is consistent with other reviews on health-related materials (Friedman & Hoffman-Goetz, 2006; Friedman & Kao, 2008). Procee met the most criteria (five of six) for being culturally sensitive for African Americans, though there was some uncertainty about where the developers retrieved the information presented as no citations were present. Otherwise, the app established African Americans as a high-risk group, addressed the perceptions of African Americans about PrCA risks, included cues to action, and was linked to organizations (i.e., BME Cancer Communities and Nottingham Trent University) who cater to African Americans.

Lastly, many of the apps violated the usability heuristics (Table 2) evaluated in this article. Specifically, 6 of the 10 heuristics, on average, scored above 1.0 (i.e., more than a minor usability error), suggesting that these heuristics were a common problem across apps. These violated heuristics included: (a) help and documentation (*M* = 2.00, *SD* = .94); (b) user control and freedom (*M* = 1.58, *SD* = .99); (c) match between system and real world (*M* = 1.46; *SD* = .99); (d) recognition rather than recall (*M* = 1.40; *SD* = 1.04); (e) visibility (*M* = 1.31, *SD* = 1.09); and (f) aesthetic and minimalist design (*M* = 1.23; *SD* = 1.24).

Similar to prior research on mobile apps for lung disease (Owens et al., 2018), usability varied greatly across the apps, but the most common violation related to the lack of instructions available within an app. Many apps did not explain their purpose on the home page nor how to use the app. Furthermore, most apps made it difficult to reverse actions, and lacked flexibility such as a back button (user control and freedom). This sometimes made navigation difficult, particularly with apps that had a deep navigation structure, required many clicks to get to deeply embedded information, or were poorly organized (match between system and real world). These violations also placed a large memory/concentration demand on users (recognition rather than recall), as it was difficult to get “lost” in the navigation structure. Lastly, few of the apps followed design principles ideal for an aging user group. Small font, low contrast, and visual noise (visibility, aesthetic, and minimalist design) can place perceptual burden on the user, making an app unnecessarily difficult for older users.

### Recommendations

On the basis of this evaluation, six key recommendations are listed below for improving the quality of commercially available PrCA education apps:

#### Apps should include information that is consistent with the latest evidence

Developers should identify organizations such as the ACS, AUA, or United States Preventive Services Taskforce to determine what content is appropriate to guide IDM for PrCA screening. For example, ACS offers evidence-based educational resources for lay persons seeking PrCA screening information, which includes the Prostate Cancer Prevention and Early Detection Guidelines. This guide provides a comprehensive overview of information that a man would need to know to engage in IDM. It is also important for developers to be cognizant of the ongoing debate about the efficacy of the PSA exam (Barry, 2009). Over the years, there have been multiple shifts in PrCA screening recommendations stemming from available evidence. Though recommendations from the ACS, AUA, and the United States Preventive Services Taskforce are largely parallel (American Cancer Society, 2016; Bibbins-Domingo et al., 2017; Carter et al., 2013), the organizations’ concurrence on IDM as a recommendation for PrCA screening didn’t occur until 2017. Prior to that, the United States Preventive Services Taskforce recommended against routine PSA screening for healthy men (Moyer, 2012). There is evidence that the varying PrCA screening recommendations has created discontinuity in health-care provider’s discussion with patients about screening (Fleshner, Carlsson, & Roobol, 2017). Therefore, it is important men are aware that not everyone agrees with PSA screening.

#### Apps should use culturally sensitive language

Although PrCA incidence and mortality is far more common among African Americans than other racial groups (Siegel et al., 2018), only three apps met over half of the criteria for being culturally sensitive. Providing information that infuses the existing evidence with culture-specific perceptions of African Americans about PrCA is of critical importance to promoting IDM for PrCA screening because ethnic minorities’ cultural beliefs are highly influential and can determine whether and to what extent individuals engage in health-care behaviors (Machirori, Patch, & Metcalfe, 2018; Tucker et al., 2014), which can affect health outcomes. Prior research has also demonstrated that minorities such as African Americans prefer health information that is culturally relevant (Chan, Haynes, O’donnell, Bachino, & Vernon, 2003; Kulukulualani, Braun, & Tsark, 2008), though many existing cancer prevention materials are not tailored to this minority population (Friedman & Kao, 2008). Using an organized checklist such as the Cultural Sensitivity Checklist (used for this study) (Friedman & Hoffman-Goetz, 2006) or the Cultural Sensitivity Assessment Tool (CSAT; Guidry & Walker, 1999) can be advantageous for developers who are seeking to develop tools specifically for African Americans, as both checklists take into account whether, and to what extent, a health education resource has culturally sensitive content and imagery.

#### App developers should be aware of the implications of tone and framing of content

The majority of apps were neutral in tone, with the exception to one app that was categorized as pro-screening. Employing a neutral tone in PrCA screening interventions may be particularly important for helping users to weigh the pros and cons of PrCA screening. A neutral tone is also more congruent with current screening recommendations, which neither encourage nor discourage screening but instead support men’s engagement in IDM with a health-care provider. Developers should also consider the limitations of their apps for promoting IDM given the absence of framing in their messaging. Instead of only providing a technical description of the different types of screening exams available, developers may use gain/loss frames to discuss the costs and/or benefits of PrCA screening, which may lead to more men having conversations with their health-care provider about PrCA screening.

#### Apps should be interactive

Most apps reviewed had little or no interactivity. Previous research (Heffernan et al., 2016; Rubinelli et al., 2013; West, Belvedere, Andreasen, Frandsen, Hall, & Crookston, 2017) recognizes that an app’s engagement of a user through strategies that require interaction between the app and the user is a significant predictor of whether the app will effectively promote behavior change. Interactive apps that allow users to weigh the pros and cons of PrCA screening may be an effective way to educate users while simultaneously requiring them to assess information that can facilitate IDM. Some interactive features that have been used in PrCA interventions include risk calculators (Pereira-Azevedo & Venderbos, 2018), decision support tools (Allen, Mohllajee, Shelton, Drake, & Mars, 2009; Allen et al., 2010), social matching and question/answer exercises (Owens, Friedman, Brandt, Bernhardt, & Hébert, 2015; Volk et al., 2008), and interactive role play with embodied conversational agents (Owens et al., 2015). The implementation of these features within an app should, however, be guided by the needs and preferences of the target population and the appropriate theory.

#### Apps should be usable

Because PrCA most often affects adults who are middle aged or older, developers should choose a framework that will ensure their app is amenable to an aging population. Older adults and those with lower incomes are more likely to have lower eHealth literacy (Neter & Brainin, 2012), which is defined as “the ability to seek, find, understand and appraise health information from electronic sources and apply knowledge gained to addressing or solving a health problem” (Norman & Skinner, 2006, p. 9). To enhance e-Health literacy, Norman and Skinner (2006) stress the importance of designing electronic interfaces that are easier to use that can negate some of the barriers associated with seeking and finding health information through an electronic source (e.g., app), thereby providing access to health content. The extent to which the content can be understood or appraised is highly dependent on factors such as readability and general health literacy, both discussed earlier in this review. There are multiple evidence-based principles for designing interfaces among older adults. For example, Czaja, Rogers, Fisk, Charness, and Sharit (2009) have published principles (e.g., increasing font sizes to enhance visibility) that are similar in nature to the heuristics used to evaluate the apps in this review (Nielsen, 1994) but that are even more suitable for an aging population who may experience working memory limitations, declining dexterity, and diminishing vision. For example, due to the lack of screen real-estate on a mobile device, presenting verbose and complicated sentences or using advance words (which would increase reading level) could also result in visibility issues. Therefore, succinct and readable text is highly advisable. Following these, or similar guidelines, could mitigate the many usability limitations discovered in this review, as well as increase the possibility that men will adopt a given app for regular use (Venkatesh & Bala, 2008).

#### Apps should be developed through a user-centered, collaborative design process

There are several multi-dimension challenges to building an effective app which span beyond those addressed earlier. Joorabchi, Mesbah, and Kruchten (2013) report that there are at least five major challenges faced by the app developers which are primarily related to the development and testing of apps for use on multiple platforms (i.e., iOS, Android, Windows). Specifically, each platform has different user interfaces which are guided by varying human computer interaction standards and require different program languages. Therefore, from a technical perspective, scientists should choose a developer that is familiar with the platform that is most often used by their target population. In addition, Owens (2015) also notes that using a community-based participatory design process within various stages of the standard app design cycle can potentially identify barriers or facilitators that could ultimately affect African American men’s acceptance of a PrCA app. For example, prior to the development of a PrCA app, Owens (2015) investigated those cultural practices, shared needs, and self-constructed and social representations of identity among African American men. These representations were then projected within the app through an African American avatar that was of similar age to the participants and also deemed culturally acceptable. However, it is noted that implementing a community-based participatory process during an app development cycle can greatly extend the app development timeline.

### Limitations

The review did not include an investigation of the source(s) developers may have used for their PrCA content. Therefore, there may have been non evidence-based information included within an app that was beyond the scope of this review. Despite this limitation, the review provided valuable findings about the quality of the app content for supporting IDM based on a comparison of app content with an existing evidence-based source.

## Conclusion

Few apps exist to promote informed PrCA screening decisions. Though most of these apps contained topics consistent with the existing ACS Prostate Cancer Prevention and Early Detection Guidelines, the information within these apps may not be comprehensive enough to facilitate an informed PrCA screening decision. In addition, the app content was not culturally sensitive or produced for individuals below an eighth-grade reading level. Therefore, the content may not be ideal for African Americans or accessible to those with lower education levels. Apps most often had a neutral tone in regard to PrCA screening, which is consistent with the current screening recommendations. Due to the brevity and factual nature of content presented in apps, the use of framing was rare. Furthermore, the usability of apps reviewed varied greatly with many demonstrating limitations that could make the app difficult to use for an older adult or a person or those with less technology-use experience. To enhance the accessibility of commercially available apps for promoting informed PrCA screening decisions, six key recommendations were provided. Furthermore, health practitioners should not solely recommend apps to prepare men to make informed decisions about PrCA screening because many apps fail to include pertinent information. Having incomplete information about PrCA screening can not only lead an uninformed screening decision but also result in other premature actions (e.g., biopsy) with potentially life changing consequences. Therefore, practitioners should recommend PrCA screening apps only in conjunction with other evidence-based, culturally-sensitive resources.
